# Genetic characterization of novel class 1 Integrons In0, In1069 and In1287 to In1290, and the inference of In1069-associated integron evolution in *Enterobacteriaceae*

**DOI:** 10.1186/s13756-017-0241-9

**Published:** 2017-08-22

**Authors:** Dongguo Wang, Jianfeng Zhu, Kaiyu Zhou, Jiayu Chen, Zhe Yin, Jiao Feng, Liman Ma, Dongsheng Zhou

**Affiliations:** 1 Department of Clinical Laboratory Medicine, Taizhou Municipal Hospital affiliated with Taizhou University and the Institute of Molecular Diagnostics of Taizhou University, 381 Zhongshan Eastern Road, Taizhou, Zhejiang, 318000 China; 2 Department of Clinical Laboratory Medicine, Yinzhou No. 2 Hospital of Ningbo, 1 Qianhe Road, Ningbo, Zhejiang, 315100 China; 3 Department of the Neurosurgery, Taizhou Municipal Hospital affiliated to Medical College of Taizhou University, 381 Zhongshan Eastern Road, Taizhou, Zhejiang, 318000 China; 4 Basic Department, Medical College of Shaoxing University, 508 Huancheng western Road, Shaoxing, Zhejiang, 312099 China; 5 State Key Laboratory of Pathogen and Biosecurity, Beijing Institute of Microbiology and Epidemiology, No. 20, Dongdajie, Fengtai, Beijing, 100071 China; 6 Department of Laboratory medicine, Medical College of Taizhou University, 1139 Shifu Avenue, Taizhou, Zhejiang, 318000 China

**Keywords:** Class 1 integron, Gene cassette, *bla*_KPC-2_, *bla*_IMP-30_, *Enterobacteriaceae*

## Abstract

**Background:**

This study aims to characterize genetically related class 1 integrons In1069, In893 and In1287 to In1290, and to further propose a scheme of stepwise integration or excision of individual gene cassettes (GCs) to generation of these integron variations.

**Methods:**

Six of 139 non-redundant *Enterobacteriaceae* strains were studied by bacterial antimicrobial susceptibility testing, detection of carbapenemase activity, and integron sequencing and sequence comparison.

**Results:**

Six novel class 1 integrons, In0, In1069, and In1287 to In1290, together with the previously characterized In893, were determined from the above strains. An unusual *bla*_KPC-2_-carrying In0 and the *bla*_IMP-30_-carrying In1069 coexists in a single isolate of *Escherichia coli*. In0 contains a PcH1 promoter and a truncated *aacA4’-3* gene cassette (GC*aacA4’-3*), as well as a *bla*_KPC-2_-containing region of Tn*6296* integrated between PcH1 and GC*aacA4’-3*. In1069 carries GC*bla*_IMP-30_ and GC*aacA4’-3* in this order. The other five integrons, In893 and In1287 to In1290, are genetically related to In1069, and all possess a core GC*aacA4’-3*. The integration or excision of one or more individual gene cassettes, such as GC*bla*_IMP-30_, GC*aadA16*, GC*catB3*, GC*arr3* and GC*dfrA27*, upstream or downstream of GC*aacA4’-3* generates various gene cassettes arrays among these five integrons.

**Conclusions:**

These findings provide the insight into stepwise and parallel evolution of In1069-associated integron variations likely under antibiotic selection pressure in clinical settings.

**Electronic supplementary material:**

The online version of this article (doi:10.1186/s13756-017-0241-9) contains supplementary material, which is available to authorized users.

## Background

Integrons are genetic elements containing a site-specific recombination system capable of integrating, exchanging, and expressing gene cassettes (GCs). Each GC is composed of an exogenous and often promoterless gene together with a recombination site, *attC* [1–5]. The recombination system possesses an integrase gene, *intI*, needed for site-specific recombination, an adjacent recombination site, *attI*, recognized by the *IntI* integrase, and a promoter, Pc, located upstream of *attI* and necessary for efficient transcription and expression of GCs [1–5]. The *attC* site is also recognized by *IntI*, and recombination between *attC* and *attI* leads to the addition and exchange of GCs and further generation of a multi-GC array within the integron structure [1–5].

Based on the amino acid sequences of *IntI* integrase, integrons can be divided into different classes, with those carrying *intI1* defined as class 1, *intI2* as class 2, *intI3* as class 3, and so on. Class 1 integrons are the most common type present among *Enterobacteriaceae* isolates [6–8]. Class 1 integrons constitute a substantial reservoir of resistance genes that confer a selective advantage upon strong selection pressure imposed by human use of antimicrobial compounds, leading to the horizontal transfer of integron-carrying resistance markers from the community to hospitals and the development of multidrug resistance (MDR) among *Enterobacteriaceae*, independent of species or isolate origin [6–8].

The ancestors of class 1 integrons are not considered to be mobile elements, and the connection of class 1 integrons with Tn*402* with a complete *tniABQC* transposition module generates a hybrid structure flanked by the 25 bp terminal inverted repeat initial (IRi) and inverted repeat terminal (IRt), making class 1 integrons capable of self-mobility [1–3]. The capture and formation of a quaternary ammonium compound resistance (*qacEΔ1*)-sulphonamide resistance (*sul1*)-*orf5* region occurs immediately downstream of the GC array [1–3]. Eventually, class 1integrons manifest as a prototype structure organized in order of IRi, a 5′-conserved segment (5′-CS: *intI*-*attI*), a central variable region (the GC array), a 3′-conserved segment (3′-CS: *qacEΔ1*-*sul1*-*orf5*-*tniABQC*), and IRt [1–3]. Most class 1 integrons from clinical contexts carry modifications at their 5′ and 3′ ends, especially partial or complete deletions of the *tniABQC* module of Tn*402*, which impairs their mobility [4, 5]. These integrons are often inserted within mobile DNA elements such as plasmids and transposons, facilitating their rapid spread in the community and within hospitals [4, 5].

This work presents the sequences of six novel class 1 integrons, In0, In1069 and In1287 to In1290, together with the previously characterized In893. These integrons were obtained from clinical *Escherichia coli*, *Klebsiella pneumoniae*, and *Enterobacter cloacae* isolates. The *bla*_KPC-2_-carrying unusual In0 and the *bla*_IMP-30_-carrying In1069 coexist in a single *Escherichia coli* isolate. The detailed genetic characterization of genetically related integrons In1069, In893 and In1287 to In1290 denotes a scheme of stepwise integration or excision of individual GCs to generate these integron variations.

## Methods

### Bacterial isolates and identification

A total of 139 non-redundant *Enterobacteriaceae* strains, including *Escherichia coli* EC6335, *Escherichia coli* EC4212, *Klebsiella pneumoniae* KP1262, *Enterobacter cloacae* ECL2236, *Escherichia coli* EC7328 and *Klebsiella pneumoniae* KP5325, were recovered from hospitalized patients with nosocomial infections in a teaching hospital of Taizhou, China, from January 2014 to September 2015. Bacterial species were identified by 16S rRNA gene sequencing [9]. All DNA markers listed in Table 1 were screened by PCR amplification (ThermoFisher scientific, USA) using the listed primers, followed by amplicon sequencing on an ABI 3730 Sequencer (ThermoFisher scientific, USA). PCR was run for 3 min at 94 °C followed by 30 cycles of 1 min of denaturing at 94 °C and annealing at 50 to 59 °C according to various primers, with a final elongation of 10 min at 72 °C on Life Veriti® PCR machine (Invitrogen, USA). The total reaction volume was 20 μL containing 4 μL 5 × PCR buffer, 0.4 μL of 10 mM dNTPs, 1 μL each of 10 μM primers and 0.2 μL Polymerase, with nuclease-free water filled up to 20 μL. PCR amplification and amplicon sequencing were run in according with operation manual.

**Table 1:** Oligonucleotide primers used in this study

Target	Primer	Primer sequence (5′-3′)	Amplicon length (bp)	Reference
*bla*_KPC_	KPC-F	GTATCGCCGTCTAGTTCTGC	637	[26]
	KPC-R	GGTCGTGTTTCCCTTTAGCC		
*bla*_IMP_	IMP-F	GGAATAGAGTGGCTTAAYTCTC	232	[26]
	IMP-R	GGTTTAAYAAAACAACCACC		
*bla*_NDM_	NDM-F	GGTTTGGCGATCTGGTTTTC	621	[26]
	NDM-R	CGGAATGGCTCATCACGATC		
*bla*_VIM_	VIM-F	GATGGTGTTTGGTCGCATA	390	[26]
	VIM-R	CGAATGCGCAGCACCAG		
*bla*_OXA-23_	OXA-23-F	GATCGGATTGGAGAACCAGA	501	[27]
	OXA-23-R	ATTTCTGACCGCATTTCCAT		
*bla*_OXA-48_	OXA-48-F	TTGGTGGCATCGATTATCGG	744	[28]
	OXA-48-R	GAGCACTTCTTTTGTGATGGC		
*bla*_OXA-58_	OXA-58-F	AAGTATTGGGGCTTGTGCTG	599	[27]
	OXA-58-R	CCCCTCTGCGCTCTACATAC		
*aacA4*	aacA4-F	TATGAGTGGCTAAATCGAT	395	[23]
	aacA4-R	CCCGCTTTCTCGTAGCA		
*intI1*	intI1-F	GCTGAAAGGTCTGGTCATAC	515	This study
	intI1-R	GTTCTTCTACGGCAAGGTG		
*tniR*	tniR-F	CCAGGGTTGGCTGCTTGC	375	This study
	tniR-R1	ATCGCCCACCTTGGTTTCC		
*intI1* to *tniR*	intI1-F	GCTGAAAGGTCTGGTCATAC	Variable	This study
	tniR-R2	ACGCTGATCGTGTGGAAG		

### Integron cloning and sequencing

For cloning of In0 and In1069, the *bla*_KPC_- and *bla*_IMP_- positive strain EC6335 was identified by PCR. Plasmid DNA was then isolated from this strain using the AxyPrep Plasmid Miniprep kit (Axygen, USA), digested with *Bam*HI, and ligated into the cloning vector pMD19-T. This was transformed into host bacteria *Escherichia coli* TOP10, which were screened for *bla*_KPC_- or *bla*_IMP_- positivity. Inserts within recombinant pMD19-T vectors from the above transformants were sequenced using the primer walking method. For cloning of In893 and In1287 to In1290, the strains EC4212, KP1262, ECL2236, EC7328, and KP5325, each co-harboring the three genes *aacA4*, *intI1*, and *tniR*, were identified by PCR, then DNA fragments were amplified from these strains using the primer pair *intI1*-F/*tniR*-R2 and subsequently sequenced as above.

### Sequence annotation and comparison

Open reading frames were predicted using RAST 2.0 [10] combined with BLASTP/BLASTN [11] searches against the UniProtKB/Swiss-Prot database [12] and the RefSeq database [13]. Annotation of resistance genes, mobile elements, and other features was carried out using the online databases including CARD [14], ResFinder [15], ISfinder [16] and INTEGRALL [17]. Multiple and pairwise sequence comparisons were performed using MUSCLE 3.8.31 [18] and BLASTN, respectively. Gene organization diagrams were drawn in Inkscape 0.48.1 (https://inkscape.org).

### Detection of carbapenemase activity

The activity of class A/B/D carbapenemases in bacterial cell extracts was determined via a modified Carba NP test [19]. Overnight bacterial cell cultures in MH broth were diluted 1: 100 into 3 mL of fresh MH broth, and bacteria were allowed to grow at 37 °C with shaking at 200 rpm to reach an OD600 of 1.0 to 1.4. If required, ampicillin was used at 200 μg/mL. Bacterial cells were harvested from 2 mL of the above culture, and washed twice with 20 mM Tris-HCl (pH 7.8). Cell pellets were resuspended in 500 μL of 20 mM Tris-HCl (pH 7.8), and lysed by sonication, followed by centrifugation at 10,000×*g* at 4 °C for 5 min. A total of 50 μL of the supernatant (the enzymatic bacterial suspension) was separately mixed with 50 μL each of substrates I to V: 50 μL supernatant was added to 50 μL substrate I, then separately 50 μL supernatant was added to 50 μL substrate II, and so on; followed by incubation at 37 °C for a maximum of 2 h. Substrate I: 0.054% phenol red plus 0.1 mM ZnSO4 (pH 7.8); substrate II: 0.054% phenol red plus 0.1 mM ZnSO4 (pH 7.8), and 0.6 mg/μL imipenem; substrate III: 0.054% phenol red plus 0.1 mM ZnSO4 (pH 7.8), 0.6 mg/μL imipenem, and 0.8 mg/μL tazobactam; substrate IV: 0.054% phenol red plus 0.1 mM ZnSO4 (pH 7.8), 0.6 mg/μL imipenem, and 3 mM EDTA (pH 7.8); substrate V: 0.054% phenol red plus 0.1 mM ZnSO4 (pH 7.8), 0.6 mg/μL imipenem, 0.8 mg/μL tazobactam, and 3 mM EDTA (pH 7.8).

### Bacterial antimicrobial susceptibility testing

Bacterial antimicrobial susceptibility was tested by the MicroScan broth dilution method (MicroScan, USA) and interpreted according to Clinical and Laboratory Standards Institute (CLSI) guidelines [20].

### Nucleotide sequence accession numbers

The sequences of In0, In1069, In893, and In1287 to In1290 were deposited in GenBank under accession numbers KP870110, KM589497, KX434463, KX371912, and KX387648 to KX387650, respectively.

## Results

### Clinical bacterial isolates containing integrons

**Fig. 1:**
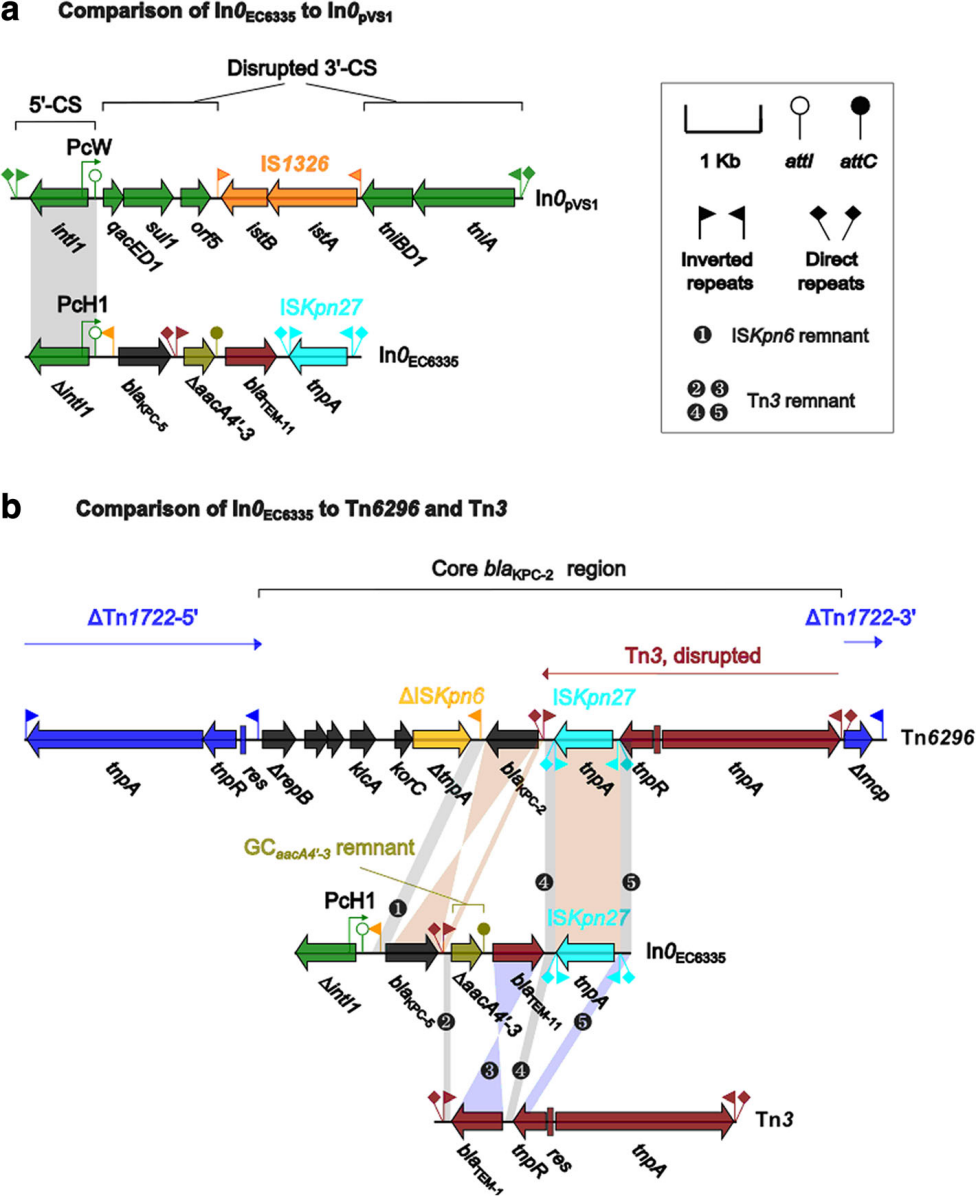
Genetic structure of In0_EC6335_ and comparison with related regions. Genes are denoted by arrows and colored according to gene function classification. Shaded areas denote regions of homology (>95% nucleotide identity). (**a**) Comparison of In0EC6335 to InpVS1; (**b**) Comparison of In0EC6335 to Tn6296 and Tn3

**Fig. 2:**
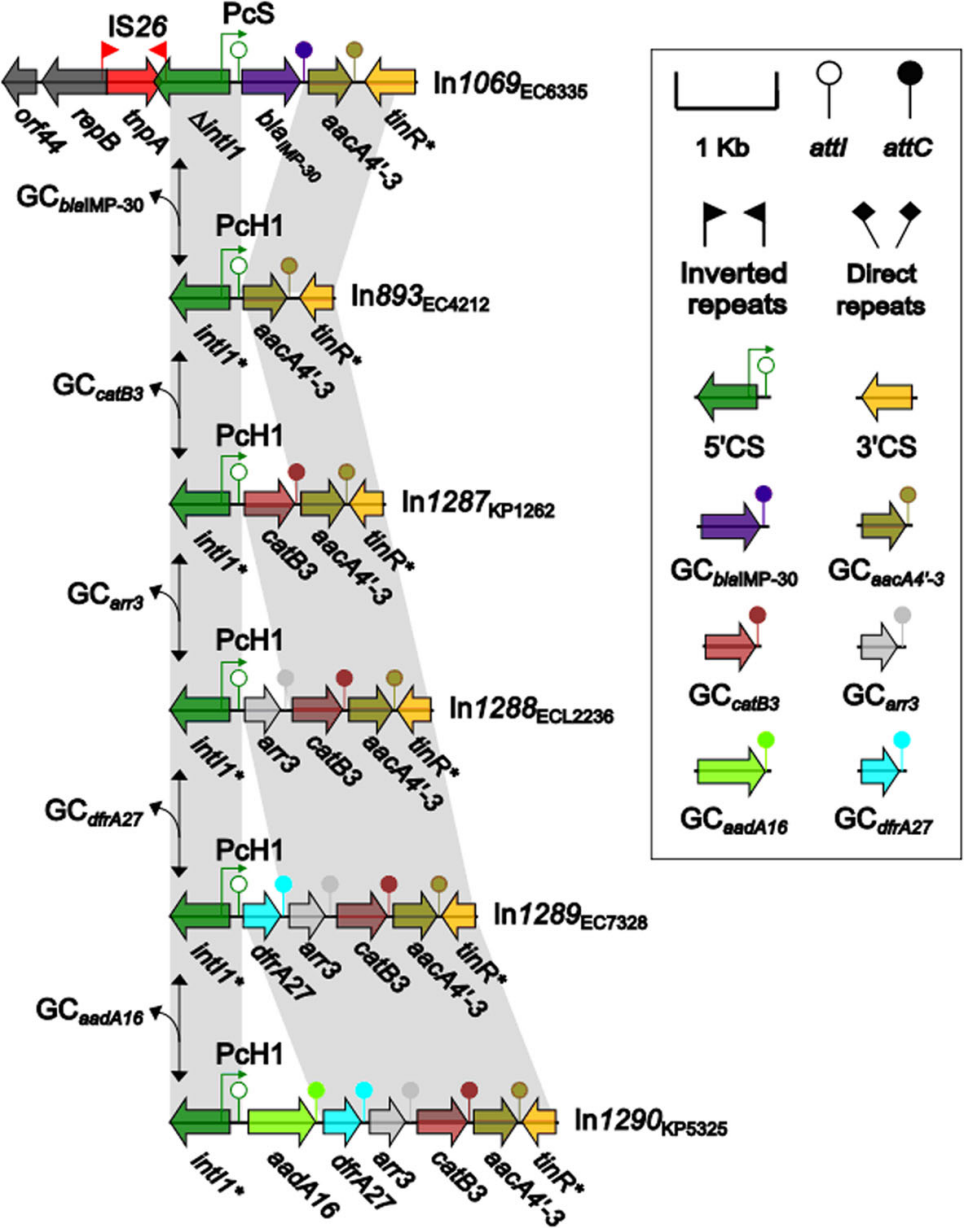
Genetic structures and proposed evolutionary history of In1069 and related integrons. Genes are denoted by arrows and colored according to gene function classification. Shaded areas denote regions of homology (>95% nucleotide identity). Double-headed arrows indicate the addition or excision of relevant gene cassttes. Asterisks denote the partially sequenced coding regions of relevant genes

*Escherichia coli* EC6335, *Escherichia coli* EC4212, *Klebsiella pneumoniae* KP1262, *Enterobacter cloacae* ECL2236, *Escherichia coli* EC7328, and *Klebsiella pneumoniae* KP5325 were determind to harbor class 1 intergrons In0 and In1069, In893, In1287, In1288, In1289, and In1290 (see Additional file 1), respetively. Modular stuctures and sequence comprasion of these integrons and related reference sequences were showed in Figs. 1 and 2. These strains were isolated from diffrent patients from the single hospital.

A 53-year-old male with vomiting, high fever, and septic shock was admitted in the hospital in August 2015. The patient had underlying diabetes, and complained of an infected leg wound. Large doses of vasoactive drugs were needed to maintain his blood pressure, but empirical intravenous antimicrobial administration of teicoplanin plus meropenem was ineffective. Carbapenem-nonsusceptible *Escherichia coli* EC6335 was subsequently isolated from the wound secretions. The patient was switched to intravenous administration of levofloxacin plus fluconazole based on antimicrobial susceptibility test results. His symptoms associated with infection progressively improved and he was discharged after 10 days of antimicrobial treatment.

*Klebsiella pneumoniae* KP1262 and *Escherichia coli* EC4212 were isolated from the blood specimens in the infection unit in July 2015. Both patients complained of suffering high fevers for two to three days, and were diagnosed to have bacteriaemia. *Enterobacter cloacae* ECL2236 was isolated from the sputum specimens in the respiration medicine unit in January 2014. *Escherichia coli* EC7328 was recovered from the urine specimens in the urinary surgery ward in September 2015. *Klebsiella pneumoniae* KP5325 was a cultivated from the sputum in the neurosurgery unit in May 2014. All of these three patients were treated by intravenous administration of ceftriaxone according to the antimicrobial susceptibility test results, and their symptoms associated with infections were gradually recovered after several days of treatments.

### *Coexistence of In0 and In1069 in Escherichia coli EC6335*

PCR screening indicated the presence of *bla*KPC and *bla*IMP, but none of the other tested carbapenemase genes *bla*NDM, *bla*VIM, *bla*OXA-23, *bla*OXA-48, or *bla*OXA-58, in strain EC6335. Further cloning and sequencing disclosed that EC6335 harbored two novel class 1 integrons: an unusual *bla*KPC-2-carrying In0EC6335 and a *bla*IMP-30-carrying In1069. In0EC6335 and In1069 (see Additional file 2) were present in two different *Bam*HI-digested EC6335 DNA fragments, which were independently cloned into pMD19-T and transferred into *Escherichia coli* TOP10, generating the *Escherichia coli* transformants In0-TOP10 and In1069-TOP10, respectively.

Strains In0-TOP10 and In1069-TOP10 have class A and B carbapenemase activities, respectively, while EC6335 appears to have class A + B activity (data not shown). All the above strains were resistant to the cephalosporin, carbapenem, and aminoglycoside drugs tested but remained susceptible to the fluoroquinolone drugs tested (Table 2). EC6335 displayed much higher levels of resistance to cephalosporins/carbapenems than In0-TOP10 and In1069-TOP10 (Table 2), which was consistent with the fact that EC6335 harbors two carbapenemase genes, blaKPC-2 and blaIMP-30, while In0-TOP10 and In1069-TOP10 carry only one gene, *bla*KPC-2 or *bla*IMP-30, respectively.

**Table 2:** Antimicrobial drug susceptibility profiles

Category	Antibiotics	MIC (mg/L)/antimicrobial susceptibility			
		EC6335 (In0+ In1069)	In0-TOP10 (In0)	In1069-TOP10 (In1069)	TOP10
Cephalosporins	Cefazolin	512/R	16/R	256/R	1/S
	Ceftazidime	256/R	4/R	128/R	0.5/S
	Ceftriaxone	256/R	8/R	128/R	0.5/S
Carbapenems	Ertapenem	16R	8/R	8/R	0.5/S
	Meropenem	16/R	8/R	8/R	0.5/S
	Imipenem	8/R	4/R	4/R	0.25/S
Aminoglycosides	Netilmicin	256/R	64/R	16/R	2/S
	Tobramycin	128/R	32/R	16/R	0.025/S
	Amikacin	512/R	128/R	32/R	1/S
Fluoroquinolones	Norfloxacin	0.10/S	0.10/S	0.05/S	0.05/S
	Ofloxacin	0.005/S	0.005/S	0.003/S	0.003/S
	Ciprofloxacin	0.25/S	0.25/S	0.125/S	0.0125/S

*S* sensitive; *R* resistant

## Discussion

### Genetic futures of novel In0_EC6335_

In*0*_EC6335_ differs dramatically from the prototype In*0*_pVS1_ (accession number U49101) from the *Pseudomonas aeruginosa* plasmid pVS1, and they only overlap each other by a majority region of 5′-CS (Fig. 1). In*0*_pVS1_ has a weak PcW promoter and an unoccupied *attI* site with no gene cassettes, thereby representing an ancestor of more complex integrons [21]. In*0*_pVS1_ possesses an intact 5′-CS, whereas its 3′-CS is disrupted by the insertion of IS*1326*, leading to the truncation of *tniABQC* of Tn*402* into *tniA*-*tniBD1* [21].

In*0*_EC6335_ contains a strong PcH1 promoter and a truncated GC*aacA4’-3* composed of *∆aacA4’-3* and *attC*_aacA4_; a *bla*_KPC-2_-containing region of Tn*6296* has been integrated between the PcH1 promoter and the truncated GC*aacA4’-3*, and located downstream of the GC*aacA4’-3* remnant are a *bla*_TEM-1_-containing region of Tn*6296* and an IS*Kpn27*- containing region from Tn*6296* (Fig. 1). *AacA4’-3* (555 bp in length; aminoglycoside resistance) is a derivative of the reference *aacA4* gene (accession number AF034958), and encodes the variations Asn5Thr and Leu102Ser compared with *aacA4*. The *∆aacA4’-3* gene of In*0*_EC6335_ has a 36 bp deletion at its 5′ end. It is most likely that In*0*_EC6335_ originates from a GC*aacA4’-3*-carrying integron that has recombined with Tn*6296*, which is one of the major mobile platforms of *bla*_KPC_ genes in China [22–24], and Tn*3* as a major mobile platform of *bla*_TEM-1_ [22–24].

### Inferred evolution of In1069 and related integrons in Enterobacteriae

In*1069* carries two GCs, GC*bla*_IMP-30_ (carbapenem resistance) and GC*aacA4’-3*, in this order, and its *intI1* gene is truncated because of the connection of IS*26* at its 3′ end. We also determined the sequences of four genetically related integrons, In*893* and In*1287* to In*1290*, from three different *Enterobacteriaceae* species: *Escherichia coli*, *Klebsiella pneumoniae*, and *Enterobacter cloacae* (Fig. 2). In*1069* and the other four integrons carry PcS and PcH1, which are both strong promoters known to drive GC expression [21].

These integrons appear to have conserved 5′-CS and 3′-CS *(tni of* Tn*402*), and possess a core GC*aacA4’-3* in which the *attC* site has an unusual 9 bp deletion (CCCTTCCAT) (Fig. 2). Five more GCs, including GC*bla*_IMP-30_, GC*aadA16* (aminoglycoside resistance), GC*catB3* (phenicol resistance), *GCarr3*, and GC*dfrA27* (rifampin resistance), are also present in these integrons. The integration/excision of one or more of these GCs upstream/downstream of the GC*aacA4’-3* core generates various organizations of GC arrays, which are mediated by the *IntI*-based, *attC*-recognizing site-specific recombination system [25]. In*893* seems to represent the most primitive form of these integrons. In*893*, In*1069*, and In*1287* carry one or two different resistance markers, while In*1288* to In*1290* have evolved to capture the determinants for at least three different classes of antibiotics, mostly likely conferring MDR. These findings provide the insight into stepwise and parallel evolution of In*1069*-associated integron variations (Fig. 2).

## Conclusions

Excessive use of antibiotics causes the spread of MDR *Enterobacteriaceae* strains in clinical settings, most of which harbor class 1 integrons. The characterization of novel class 1 integrons In0, In1069 and In1287 to In1290 denotes a step-by-step and parallel evolution scheme involving massive genetic changes in integron GC arrays under high levels of antibiotic selection pressure in clinical settings.

## Additional files

Additional file 1: Sequence analysis for In0, In1069, In893, and In1287 to In1290, respetively. (ZIP 65 kb)

Additional file 2: Integron analysis for In0 and In1069. (XLS 33 kb)

## Abbreviations

3′-CS: 3′-conserved segment
5′-CS: 5′-conserved segment
GC: gene cassette
MDR: multidrug resistance
MIC: minimum inhibitory concentration
PCR: polymerase chain reaction

## References

[1] Partridge SR, Tsafnat G, Coiera E, Iredell JR (2009). Gene cassettes and cassette arrays in mobile resistance integrons. FEMS Microbiol Rev.

[2] Domingues S, da Silva GJ, Nielsen KM (2012). Integrons: vehicles and pathways for horizontal dissemination in bacteria. Mobile genetic elements.

[3] Gillings MR (2014). Integrons: past, present, and future. Microbiol Mol Biol Rev.

[4] Rowe-Magnus DA, Guerout AM, Mazel D (2002). Bacterial resistance evolution by recruitment of super-integron gene cassettes. Mol Microbiol.

[5] Gillings M, Boucher Y, Labbate M, Holmes A, Krishnan S, Holley M, Stokes HW (2008). The evolution of class 1 integrons and the rise of antibiotic resistance. J Bacteriol.

[6] Leverstein-van Hall MA, Blok HE M, Donders AR T, Paauw A, Fluit AC, Verhoef J (2003). Multidrug resistance among Enterobacteriaceae is strongly associated with the presence of integrons and is independent of species or isolate origin. J Infect Dis.

[7] Leverstein-Van Hall MA, Paauw A, Box AT, Blok HE, Verhoef J, Fluit AC (2002). Presence of integron-associated resistance in the community is widespread and contributes to multidrug resistance in the hospital. J Clin Microbiol.

[8] White PA, McIver CJ, Rawlinson WD (2001). Integrons and gene cassettes in the enterobacteriaceae. Antimicrob Agents Chemother.

[9] Frank JA, Reich CI, Sharma S, Weisbaum JS, Wilson BA, Olsen GJ (2008). Critical evaluation of two primers commonly used for amplification of bacterial 16S rRNA genes. Appl Environ Microbiol.

[10] Brettin T, Davis JJ, Disz T, Edwards RA, Gerdes S, Olsen GJ, Olson R, Overbeek R, Parrello B, Pusch GD (2015). RASTtk: a modular and extensible implementation of the RAST algorithm for building custom annotation pipelines and annotating batches of genomes. Sci Rep.

[11] Boratyn GM, Camacho C, Cooper PS, Coulouris G, Fong A, Ma N, Madden TL, Matten WT, McGinnis SD, Merezhuk Y et al: BLAST: a more efficient report with usability improvements. *Nucleic Acids Res* 2013, 41(Web Server issue):W29–33. 10.1093/nar/gkt282 PMC3692093 23609542

[12] Boutet E, Lieberherr D, Tognolli M, Schneider M, Bansal P, Bridge AJ, Poux S, Bougueleret L, Xenarios I (2016). UniProtKB/Swiss-Prot, the manually annotated section of the UniProt KnowledgeBase: how to use the entry view. Methods Mol Biol.

[13] O'Leary NA, Wright MW, Brister JR, Ciufo S, Haddad D, McVeigh R, Rajput B, Robbertse B, Smith-White B, Ako-Adjei D (2016). Reference sequence (RefSeq) database at NCBI: current status, taxonomic expansion, and functional annotation. Nucleic Acids Res.

[14] Jia B, Raphenya AR, Alcock B, Waglechner N, Guo P, Tsang KK, Lago BA, Dave BM, Pereira S, Sharma AN, et al. CARD 2017: expansion and model-centric curation of the comprehensive antibiotic resistance database. Nucleic Acids Res. 2016; 10.1093/nar/gkw1004 PMC5210516 27789705

[15] Zankari E, Hasman H, Cosentino S, Vestergaard M, Rasmussen S, Lund O, Aarestrup FM, Larsen MV (2012). Identification of acquired antimicrobial resistance genes. J Antimicrob Chemother.

[16] Siguier P, Perochon J, Lestrade L, Mahillon J, Chandler M (2006). ISfinder: the reference centre for bacterial insertion sequences. Nucleic Acids Res.

[17] Moura A, Soares M, Pereira C, Leitao N, Henriques I, Correia A (2009). INTEGRALL: a database and search engine for integrons, integrases and gene cassettes. Bioinformatics.

[18] Edgar RC (2004). MUSCLE: multiple sequence alignment with high accuracy and high throughput. Nucleic Acids Res.

[19] Chen Z, Li H, Feng J, Li Y, Chen X, Guo X, Chen W, Wang L, Lin L, Yang H (2015). NDM-1 encoded by a pNDM-BJ01-like plasmid p3SP-NDM in clinical Enterobacter Aerogenes. Front Microbiol.

[20] CLSI (2015). Performance standards for antimicrobial susceptibility testing: twenty-fifth informational supplement M100-S25.

[21] Bissonnette L, Roy PH (1992). Characterization of In0 of Pseudomonas Aeruginosa plasmid pVS1, an ancestor of integrons of multiresistance plasmids and transposons of gram-negative bacteria. J Bacteriol.

[22] Wang L, Fang H, Feng J, Yin Z, Xie X, Zhu X, Wang J, Chen W, Yang R, Du H (2015). Complete sequences of KPC-2-encoding plasmid p628-KPC and CTX-M-55-encoding p628-CTXM coexisted in Klebsiella Pneumoniae. Front Microbiol.

[23] Feng J, Qiu Y, Yin Z, Chen W, Yang H, Yang W, Wang J, Gao Y, Zhou D (2015). Coexistence of a novel KPC-2-encoding MDR plasmid and an NDM-1-encoding pNDM-HN380-like plasmid in a clinical isolate of Citrobacter freundii. J Antimicrob Chemother.

[24] Dai X, Zhou D, Xiong W, Feng J, Luo W, Luo G, Wang H, Sun F, Zhou X (2016). The IncP-6 plasmid p10265-KPC from Pseudomonas Aeruginosa carries a novel DeltaISEc33-associated bla KPC-2 gene cluster. Front Microbiol.

[25] Barraud O, Ploy MC (2015). Diversity of class 1 Integron gene cassette rearrangements selected under antibiotic pressure. J Bacteriol.

[26] Poirel L, Walsh TR, Cuvillier V, Nordmann P (2011). Multiplex PCR for detection of acquired carbapenemase genes. Diagn Microbiol Infect Dis.

[27] Woodford N, Ellington MJ, Coelho JM, Turton JF, Ward ME, Brown S, Amyes SG, Livermore DM (2006). Multiplex PCR for genes encoding prevalent OXA carbapenemases in Acinetobacter spp. Int J Antimicrob Agents.

[28] Poirel L, Potron A, Nordmann P (2012). OXA-48-like carbapenemases: the phantom menace. J Antimicrob Chemother.

