# Supplementary material for: Genetic characterization of novel class 1 Integrons In0, In1069 and In1287 to In1290, and the inference of In1069-associated integron evolution in Enterobacteriaceae
Source: Antimicrob Resist Infect Control. 2017 Aug 22;6:84. doi: 10.1186/s13756-017-0241-9 (PMC5567636; doi:10.1186/s13756-017-0241-9)
Supplement: Supplementary file 1 — Sequence analysis for In0, In1069, In893, and In1287 to In1290, respetively. (ZIP 65 kb) [file 13756_2017_241_MOESM1_ESM.zip › In1287.docx]

>In1287_K.pneumoniae_KP1262

ATGTGATGGCGACGCACGACACCGCTCCGTGGATCCGAATGCGTGTGCTGCGCAAAAACCCAGAACCACGGCCAGGAATGCCCGGGGTCGCGCGGATACTTCCGCTCAAGGGCGTCGGGAAGCGCAACGCCGCTGCGGCCCTCGGCCTGGTCCTTCAGCCACCATGCCCGTGCACGCGACAGCTGCTCGCGCAGGCTGGGTGCCAAGCTCTCGGGTAACATCAAGGCCCGATCCTTGGAGCCCTTGCCCTCCCGCACGATGATCGTGCCGTGATCGAAATCCAGATCCTTGACCCGCAGTTGCAAACCCTCACTGATCCGCATGCCCGTTCCATACAGAAGCTGGGCGAACAAACGATGCTCGCCTTCCAGAAAACCGAGGATGCGAACCACTTCATCCGGGGTCAGCACCACCGGCAAGCGCCGCGACGGCCGAGGTCTTCCGATCTCCTGAAGCCAGGGCAGATCCGTGCACAGCACCTTGCCGTAGAAGAACAGCAAGGCCGCCAATGCCTGACGATGCGTGGAGACCGAAACCTTGCGCTCGTTCGCCAGCCAGGACAGAAATGCCTCGACTTCGCTGCTGCCCAAGGTTGCCGGGTGACGCACACCGTGGAAACGGATGAAGGCACGAACCCAGTGGACATAAGCCTGTTCGGTTCGTAAACTGTAATGCAAGTAGCGTATGCGCTCACGCAACTGGTCCAGAACCTTGACCGAACGCAGCGGTGGTAACGGCGCAGTGGCGGTTTTCATGGCTTGTTATGACTGTTTTTTTGTACAGTCTATGCCTCGGGCATCCAAGCAGCAAGCGCGTTACGCCGTGGGTCGATGTTTGATGTTATGGAGCAGCAACGATGTTACGCAGCAGGGCAGTCGCCCTAAAACAAAGTTAGATGCACGCAATTAGACGGCAAAGTCACAGACCGCGGGATCTCTTATGACCAACTACTTTGATAGCCCCTTCAAAGGCAAGCTGCTTTCTGAGCAAGTGAAGAACCCCAATATCAAAGTTGGGCGGTACAGCTATTACTCTGGCTACTATCATGGGCACTCATTCGATGACTGCGCACGGTATCTGTTTCCGGACCGTGATGACGTTGATAAGTTGATCATCGGTAGTTTCTGCTCTATCGGGAGTGGGGCTTCCTTTATCATGGCTGGCAATCAGGGGCATCGGTACGACTGGGCATCATCTTTCCCGTTCTTTTATATGCAGGAAGAACCTGCATTCTCAAGCGCACTCGATGCCTTCCAAAAAGCAGGTAATACTGTCATTGGCAATGACGTTTGGATCGGCTCTGAGGCAATGGTCATGCCCGGAATCAAGATCGGGCACGGTGCGGTGATAGGCAGCCGCTCGTTGGTGACAAAAGATGTGGAGCCTTACGCTATCGTTGGCGGCAATCCCGCTAAGAAGATTAAGAAACGCTTCACCGATGAGGAAATTTCATTGCTTCTGGAGATGGAGTGGTGGAATTGGTCACTGGAGAAGATCAAAGCGGCAATGCCCATGCTGTGCTCGTCTAATATTGTTGGCCTGCACAAGTATTGGCTCGAGTTTGCCGTCTAACAATTCAATCAAGCCGATGCCGCTTCGCGGCACGGCTTATTTCAGGCGTTATGCTTAGGCATCACAAAGTACAGCATCGTGACCAACAGCACCGATTCCGTCACACTGCGCCTCATGACTGAGCATGACCTTGCGATGCTCTATGAGTGGCTAAATCGATCTCATATCGTCGAGTGGTGGGGCGGAGAAGAAGCACGCCCGACACTTGCTGACGTACAGGAACAGTACTTGCCAAGCGTTTTAGCGCAAGAGTCCGTCACTCCATACATTGCAATGCTGAATGGAGAGCCGATTGGGTATGCCCAGTCGTACGTTGCTCTTGGAAGCGGGGACGGATGGTGGGAAGAAGAAACCGATCCAGGAGTACGCGGAATAGACCAGTCACTGGCGAATGCATCACAACTGGGCAAAGGCTTGGGAACCAAGCTGGTTCGAGCTCTGGTTGAGTTGCTGTTCAATGATCCCGAGGTCACCAAGATCCAAACGGACCCGTCGCCGAGCAACTTGCGAGCGATCCGATGCTACGAGAAAGCGGGGTTTGAGAGGCAAGGTACCGTAACCACCCCAGATGGTCCAGCCGTGTACATGGTTCAAACACGCCAGGCATTCGAGCGAACACGCAGTGATGCCTAACGAGGGGGACGTCCAAGGGCTGGCGCCCTTGGCCGCCCCTCATGTCAAACGATTTATGCGGCTGAACCGAGGGAGAGCAGCTTTACGCCGTCTGGCCGCAGTTCGCCCTTGGGCGACACGTGCCGGTAGAGCGTCTGCCGGGTAATCCCGAGTTCTTCGCAGAGATCGCCCACCTTGGTTTCCGGTTGCCCCATGCTGGCCATCGCCAGGCGTAGCTTGGCGGCGGTCATCTTGAAGGGGCGCCCCCCTTTCCTGCCGCGAGCGCGCGCCGAGATAAGTCCAGCGACTGTTCGCTCGGAAATCAACTCACGCTCGAACTCGGCCAGCGCGGCAAAAATACCGAACACAAGCTTGCCGGCGGCAGTCGTCGTGTCGACCGCCGCACCGTGACCGGTCAGGACCTTCAGGCCCACGCTACGCGCAGTTAGGTCGTGCACGGTGTTGATCAGGTGGCGCAGATCACGGCCAAGCCGATCGAG
